# Coarse Graining, Nonmaximal Entropy, and Power Laws

**DOI:** 10.3390/e20100737

**Published:** 2018-09-26

**Authors:** Fernando C. Pérez-Cárdenas, Lorenzo Resca, Ian L. Pegg

**Affiliations:** 1 Vitreous State Laboratory, The Catholic University of America, Washington, DC 20064, USA; 2 Department of Physics and Vitreous State Laboratory, The Catholic University of America, Washington, DC 20064, USA

**Keywords:** entropy, coarse graining, power laws, nonmaximal entropy

## Abstract

We show that coarse graining produces significant and predictable effects on the entropy of states of equilibrium when the scale of coarse graining becomes comparable to that of density fluctuations. We demonstrate that a coarse-grained entropy typically evolves toward a state of effective equilibrium with a lower value than that of the state of maximum entropy theoretically possible. The finer the coarse graining, the greater the drop in effective entropy, and the more relevant the fluctuations around that. Fundamental considerations allow us to derive a remarkable power law that relates coarse graining to the effective entropy gap. Another power law is found that precisely relates the noise range of effective entropy fluctuations to coarse graining. We test both power laws with numerical simulations based on a well-studied two-dimensional lattice gas model. As expected, the effects of these power laws diminish as our description approaches a macroscopic level, eventually disappearing in the thermodynamic limit, where the maximum entropy principle is reasserted.

## 1. Introduction

The discovery and formulation of entropy and its maximization principles, in relation to the second law of thermodynamics and the arrow of time, stand among the greatest experimental and intellectual achievements of science. From the original works of genius of Clausius, Maxwell, Thomson, Boltzmann, Gibbs, Planck, and Einstein, to later developments by von Neumann, Shannon, Jaynes, and others, principles of most probable distributions and maximal entropy have always maintained and even expanded their fundamental role [1]. Of particular interest to the present paper, we cannot fail to mention the pioneering work of Grad in relation to entropy and coarse graining [2].

While investigating from a coarse grained perspective the transition from microscopic reversibility to macroscopic irreversibility, we came across a remarkable finding: see Figures 4 and 5 of Reference [3]. Namely, the *coarse-grained* entropy of a finite lattice gas, *S*^(*c*)^, typically evolves toward an equilibrium average, $S_{equil}^{\left(c\right)}$, which remains well below the maximum entropy, $S_{max}^{\left(c\right)}$, fluctuating in time around $S_{equil}^{\left(c\right)}$ within a narrow noise range. The purpose of this paper is to demonstrate how that may generally happen and why. We will show that the formation of an entropy gap between the possible maximum entropy, $S_{max}^{\left(c\right)}$, and the effective equilibrium average entropy, $S_{equil}^{\left(c\right)}$, obeys a precise power law depending on coarse graining. Yet another coarse-graining-dependent power law rules the narrow range of the entropy fluctuations around $S_{equil}^{\left(c\right)}$.

The fundamental reasoning that underlies these power laws is the following. The maximum entropy, $S_{max}^{\left(c\right)}$, is generally associated with the greatest number of microscopic states compared with any other individual distribution. However, $S_{max}^{\left(c\right)}$ derives from a single distribution, namely that in which all particles are uniformly distributed among relatively small cells in a *coarse-grained* description of the system. As a result, that uniform distribution is relatively unlikely to occur in a coarse-grained system, given the much greater number of microscopic states associated with a multitude of distributions in which cell fluctuations occur at a finer level of coarse graining. That multitude of distributions produces a large number of equal or comparable entropies that fluctuate remarkably below $S_{max}^{\left(c\right)}$. In retrospect, such phenomena should have been expected, but they have not been unequivocally demonstrated heretofore, to the best of our knowledge. Computer simulations on lattice gases and cellular automata have been extensively performed [4,5,6,7], but perhaps coarse graining effects have not yet gained the attention that they presently deserve. A deeper account of coarse graining, theoretically started more than a century ago [8,9], is ever-increasingly relevant for experiments as a result of major advances in nanotechnology [10,11,12].

The power laws that we derive in this paper are fundamental, requiring no more than the assumption of normal fluctuations of particles in and out of coarse-grained cells. That may very well be a most general phenomenon, typically occurring in, but by no means limited to, systems such as ideal gases. We have extensively tested our two power laws with numerical calculations in a two-dimensional lattice gas model [3,6,7]. Consistently, power law effects diminish with larger coarse graining, and they eventually disappear in the thermodynamic limit, where the maximum entropy principle is reasserted.

## 2. Nonmaximal Equilibrium Entropy and Power Laws

Let us thus consider an ideal gas consisting of *M* identical and indistinguishable particles confined within a macroscopic cubic volume in *d* dimensions. Either practically or conceptually, we can subdivide that volume into *N_c_* identical cubic cells, having sides of length *c* each. One way to partially characterize a state of the system is to provide an ordered set of *N_c_* occupation numbers, *n_i_*, representing the number of particles in each cell. We denote that set as $\left\{n_{1},n_{2},\ldots ,n_{N_{c}}\right\}$ and we call it a distribution. A distribution provides only a coarse-grained description of the state of the system, common to many different microscopic states, because the distribution disregards features of the microscopic state at scales shorter than *c*. We have previously referred to such a distribution as a *c*-view of a certain lattice gas model, calling *c* the corresponding *coarse graining index* [3]. The larger the value of *c*, the greater our ignorance regarding the exact microscopic state that underlies precisely the system distribution at any given time.

Let $\Omega^{\left(c\right)}\left(\left\{n_{1,\;}n_{2},\ldots ,n_{N_{c}}\right\}\right)$ be the number of microscopic states, or microstates, underlying the $\left\{n_{1,\;}n_{2},\ldots ,n_{N_{c}}\right\}$ distribution. We can compute the coarse-grained entropy for that distribution using Boltzmann’s formula

(1) $$\;S^{\left(c\right)}=\;k_{B}{\ln\Omega }^{\left(c\right)},\;$$

where *k_B_* is Boltzmann’s constant. This unbiased counting is based on the assumption of equal a priori probability for all microstates.

Macroscopically, the system is presumed to have reached equilibrium when particles are uniformly distributed throughout the system. For large *c* values, corresponding to large cells capable of containing many particles, relative differences in the number of particles between different cells must indeed become negligible compared with the average number of particles in each cell, <*n*>. Thus, *S*^(*c*)^ attains its maximum value at equilibrium, namely,

(2) $$\;S^{\left(c\right)}\left\{n_{i}=<n>,\;\forall \;i\right\}=S_{max\;}^{\left(c\right)}.\;$$

For any other distribution, in which cell occupation numbers (*n_i_* values) vary from cell to cell, *S*^(*c*)^ must be smaller.

If we consider a finer coarse graining, i.e., a smaller *c* index, we expect smaller numerical values for *S*^(*c*)^. If at that scale particle fluctuations remain relatively negligible for most cells, then the entropy must fluctuate a little below its maximum value, as given in Equation (2). However, if we keep decreasing *c*, fluctuations in cell occupation numbers *n_i_* must eventually become more and more relevant. Greater relative variations of *n_i_* values from cell to cell cause the entropy *S*^(*c*)^ to decrease and narrowly fluctuate around an equilibrium average value, $S_{equil}^{\left(c\right)}$, well below $S_{max}^{\left(c\right)}$, for the following reasons.

When coarse graining eventually becomes sufficiently fine that we can detect major relative fluctuations in the number *n_i_* of particles in each cell, we expect that the single distribution in which all the particles are uniformly distributed throughout the system becomes unlikely, relative to a large set of distributions with equal or comparable but lower entropies in which the number of particles varies significantly from cell to cell. Thus, when the system reaches equilibrium, we expect that distributions realized by the system over time generate coarse-grained entropies that fluctuate in time within a certain noise range, but typically remain well below $S_{max}^{\left(c\right)}$.

Let us indeed recall that at any given time, *t*, the system is in a specific microstate. That microstate pertains to a specific distribution. As time evolves, other microstates are realized, some of which will pertain to that very same distribution, while other microstates will pertain to other distributions. The likelihood of a specific distribution is proportional to the number of microstates compatible with that distribution. Furthermore, it is easy to realize that there can be many different distributions that generate the same coarse-grained entropy, *S*^(*c*)^. For example, two distributions that differ by the exchange of two different cell occupation numbers—*n_i_* and *n_j_*, say—generate the same entropy. Thus, the most likely entropy is the one that corresponds to the largest number of microstates that underlie all possible different distributions, but only those that still generate the same entropy. In turn, the time-averaged macroscopic state of equilibrium corresponds to the greatest number of microscopic states that generate through whatever coarse-grained distributions the same equilibrium entropy, $S_{equil}^{\left(c\right)}$. Numerical simulations have indeed confirmed these expectations [3].

In order to estimate entropy fluctuations around equilibrium, let us define an average relative fluctuation δ^(c)^ in occupation numbers as follows:

(3) $$\;\delta^{\left(c\right)}=\frac{1}{N_{C}}\underset{i}{\sum }\left|\frac{n_{i}-<n>}{<n>}\right|.\;$$

This provides a global measure of cell fluctuations for any given *c*-view. It is reasonable to assume that *S*^(*c*)^ is an even function of *δ*^(*c*)^, namely that *S*^(*c*)^(*δ*^(*c*)^) = *S*^(*c*)^(−*δ*^(*c*)^), and that *S*^(*c*)^ attains its maximum at a uniform density, namely for *δ*^(*c*)^ = 0. Correspondingly, ${\frac{\partial S^{\left(c\right)}}{\partial \delta^{\left(c\right)}}]}_{\delta^{\left(c\right)}=0}=0$. Let us then expand *S*^(*c*)^ in powers of *δ*^(*c*)^ around its maximum, $S_{max}^{\left(c\right)}=S^{\left(c\right)}\left(\delta^{\left(c\right)}=0\right)$, following a standard procedure (as outlined in Section 110 of Reference [13], pp. 333–335, for example). We thus obtain

(4) $$\;S^{\left(c\right)}\left(\delta^{\left(c\right)}\right)=S^{\left(c\right)}\left(\delta^{\left(c\right)}=0\right)+\;\frac{1}{2}{\left(\frac{\partial^{2}S^{\left(c\right)}}{\partial \delta^{\left(c\right)}{}^{2}}\right)}_{\delta^{\left(c\right)}=0}\delta^{\left(c\right)}{}^{2}+\ldots \;.\;$$

This indicates the existence of an entropy gap relative to equilibrium, defined as

(5) $$\;\Delta S^{\left(c\right)}\equiv \;S_{max}^{\left(c\right)}-\;S^{\left(c\right)}\left(\delta^{\left(c\right)}\right)=\;S_{max}^{\left(c\right)}-S_{equil}^{\left(c\right)}\sim \;\delta^{\left(c\right)}{}^{2},\;$$

thus revealing proportionality to the square of the characteristic fluctuation, *δ*^(*c*)^.

Now, for an ideal gas, and in fact for any system exhibiting so-called normal fluctuations, we generally expect that

(6) $$\;\delta^{\left(c\right)}\sim \;1/\sqrt{<n>}\;.\;$$

Furthermore, the average number of particles in a cell, <*n*>, must be proportional to the size of the cell, *c^d^*, namely

(7) $$\;<n>\sim c^{d},\;$$

where *d* represents the spatial dimensionality of the system. We thus arrive at a power law of the form

(8) $$\;\Delta S^{\left(c\right)}\;\sim \;c^{-d},\;$$

which relates the entropy gap to coarse graining. Based on this essential derivation, this power law is expected to hold for any system that exhibits so-called normal fluctuations, such as given in Equation (6). That includes in particular, but is not limited to, ideal gas systems. Thus, over time, *S*^(*c*)^ is expected to fluctuate around its time average, $S^{\left(c\right)}=S_{equil}^{\left(c\right)}$, well below $S_{max}^{\left(c\right)}$.

We can also establish a power law for the standard deviation from $S_{equil}^{\left(c\right)}$ or noise range *δS*^(*c*)^ of *S*^(*c*)^ by performing a linear expansion of *δS*^(*c*)^ in *δ*^(*c*)^, namely,

(9) $$\;\delta S^{\left(c\right)}\sim {\left(\frac{\partial \left(\delta S^{\left(c\right)}\right)}{\partial \delta^{\left(c\right)}}\right)}_{\delta^{\left(c\right)}=0}\delta^{\left(c\right)}.\;$$

Thus, assuming once again normal fluctuations, as given in Equation (6), we arrive at another power law having the form

(10)  δS(c)~c−d2. 

Evidently, these two power laws for coarse-grained entropy gaps and fluctuations that we have derived are almost exclusively based on a quite general assumption of so-called “normal” fluctuations of particles in and out of coarse-grained cells, as represented in Equation (6).

## 3. Entropy Gap and Range of Fluctuations in a Lattice Gas

We extensively verified the power laws described in the previous section in a practical situation. Namely, we performed numerical simulations of the time evolution of the coarse-grained entropy for a two-dimensional square-lattice gas. The indistinguishable particles of this model obey reversible, deterministic dynamics, and can occupy only the nodes of a square lattice, with velocities of equal magnitude that can have only four directions, as specified in References [3,6,7]. To provide some illustration of our computational experiments, let us partition a square lattice into *N_c_* identical square cells, each containing *c* × *c* sites. We thus have *Z*^(*c*)^ = 4*c*^2^ single-particle states within each cell, since there are four possible velocity directions for each particle at each site. Assuming particle indistinguishability, the number of microstates compatible with any given $\left\{n_{1},n_{2},\ldots ,n_{N_{c}}\right\}$ distribution is

(11) $$\;\Omega^{\left(c\right)}=\overset{N_{c}}{\underset{i=1}{\prod }}\frac{Z^{\left(c\right)}!}{n_{i}!\left(Z^{\left(c\right)}-n_{i}\right)!}=\overset{N_{c}}{\underset{i=1}{\prod }}\frac{\left(4c^{2}\right)!}{n_{i}!\left(4c^{2}-n_{i}\right)!}\;$$

yielding [2]

(12) $$\;\frac{S^{\left(c\right)}}{k_{B}}=N_{c}\ln\left(4c^{2}\right)!-\overset{N_{c}}{\underset{i=1}{\sum }}\left\{\ln\;n_{i}!+\ln\left(4c^{2}-n_{i}\right)!\right\}.\;$$

A computer program determines uniquely the time evolution of the microstate that underlies whatever $\left\{n_{1,\;}n_{2},\ldots ,n_{N_{c}}\right\}$ distribution, thus determining uniquely the time evolution of the coarse-grained entropy, *S*^(*c*)^, given complete knowledge of the initial microstate.

**Figure 1:**
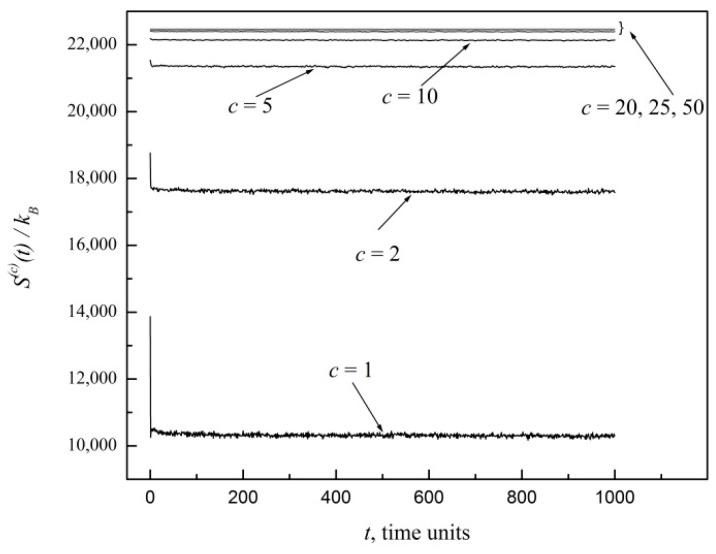
Entropy functions, *S*^(*c*)^, for a 100 × 100 site lattice gas system in which, initially, one particle is placed on each site with a random velocity direction out of four possibilities. Coarse graining indices are *c* = 1, 2, 5, 10, 20, 25, 50.

In order to test the first power law shown in Equation (8), let us consider a 100 × 100 site lattice and let us place initially one particle on each site. Furthermore, at the initial time, we randomly select one of the four possible velocity directions for each particle. For any coarse graining, the maximum entropy, $S_{max}^{\left(c\right)}$, is realized when all the 10^4^ particles are uniformly distributed among all sites, which is exactly what we have set at the initial time in our computational experiment. Starting from that $S_{max}^{\left(c\right)}$, the time evolution of the entropy, *S*^(*c*)^, is shown in Figure 1 for coarse graining indices *c* = 1, 2, 5, 10, 20, 25, 50. Notice the proximity and virtually constant values of entropies for *c* = 20, 25, 50. This is expected. However, for *c* = 1, 2, 5, there is a striking sudden drop of entropy from its initial maximum value to much lower values. Subsequently, *S*^(*c*)^ fluctuates in time within a relatively narrow noise range around its time average, hardly ever climbing back up to $S_{max}^{\left(c\right)}$. To better display the behavior, Figure 1 is limited to 1000 time units, which is more than enough for *S*^(*c*)^ to attain a state of virtual equilibrium, $S_{equil}^{\left(c\right)}$. However, we have verified with much more extensive computations that essentially the same behavior holds for much longer times. In fact, microstates with much more uniform particle distributions that may close the entropy gap for *c* = 1, 2, 5 may typically occur only on far longer time scales of the order of the Poincaré recurrence time [3].

In relation to the decreasing of $S_{equil}^{\left(c\right)}$ with decreasing *c* shown in Figure 1, we can say that this represents a numerical example of what Grad wrote in page 324 of Reference [2]: “… Intuitively, a more detailed description is capable of describing a greater degree of order and should have a smaller entropy associated with it …”.

**Figure 2:**
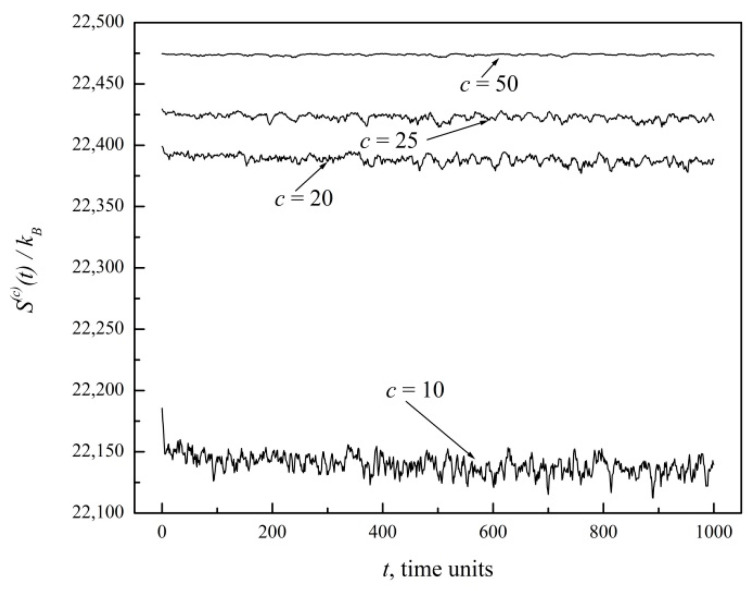
Expanded view of Figure 1 for coarse graining indices *c* = 10, 20, 25, 50.

The initial sudden drop in entropy thus reflects the fact that microstates corresponding to a uniform distribution underlying $S_{max}^{\left(c\right)}$ represent just a small fraction of all possible microstates. As *c* is increased, the size of the cells grows, relative fluctuations become less relevant, and a detailed description of the system is gradually lost in the coarser view. Thus, coarser graining results in a reduction of the entropy gap. This is further illustrated here in Figure 2, which offers an expanded view of coarse graining indices *c* = 10, 20, 25, 50. Eventually, *S*^(50)^ deviates very little from $S_{max}^{\left(50\right)}$. For larger systems and larger *c* indices, the entropy gap and relative fluctuations indeed become negligible in the thermodynamic limit at macroscopic scales, as we proved in Reference [3].

Looking again at Figure 1, it is apparent that a state of virtual equilibrium has already been attained within 50 time units from the start. So, the coarse-grained entropy *S*^(*c*)^ relaxes very quickly down to that state of virtual equilibrium, $S_{equil}^{\left(c\right)}$, and fluctuates around it within a certain noise range which becomes narrower and narrower with increasing *c* index. We may thus compute the entropy gap *ΔS*^(*c*)^ by simply subtracting from $S_{max}^{\left(c\right)}$ the time average of *S*^(*c*)^(*t*) for *t* > 100, which defines $S_{equil}^{\left(c\right)}$.

**Figure 3:**
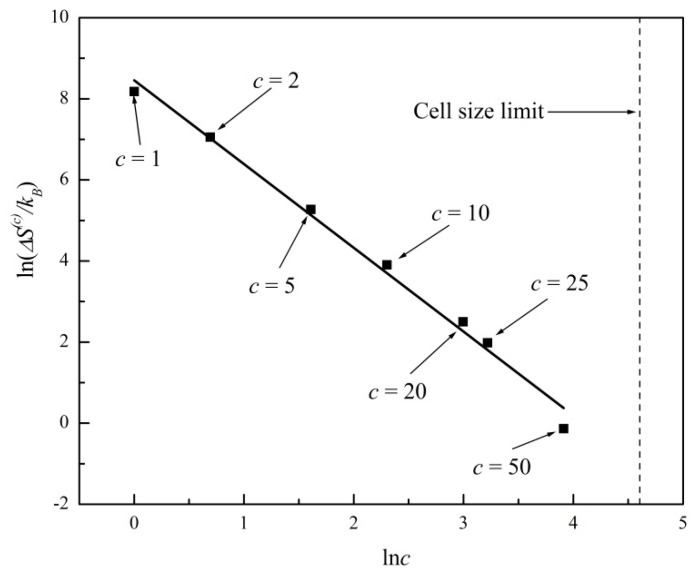
Plot of ln*ΔS*^(*c*)^ versus ln(*c*) for the entropy evolutions shown in Figure 1 and Figure 2, where *ΔS*^(*c*)^ is the entropy gap. Linear regression fit with slope −2.07 ± 0.09 (R^2^ = 0.99). *Cell size limit* corresponds to *c* = 100.

**Figure 4:**
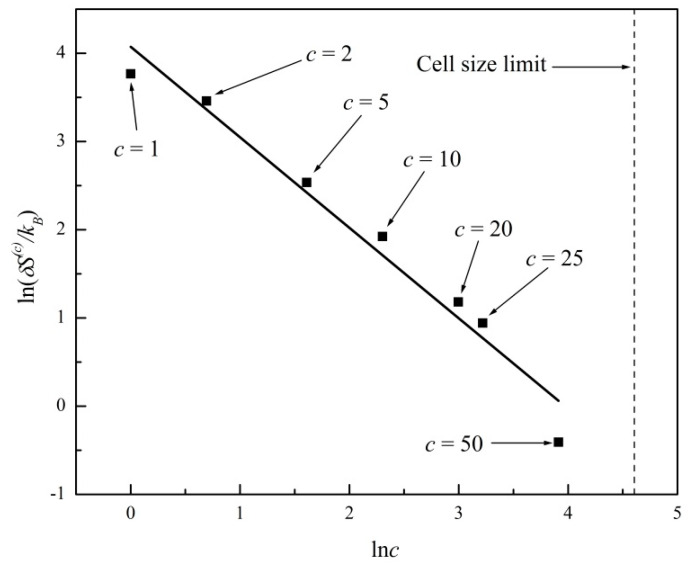
Plot of ln*δS*^(*c*)^ versus ln(*c*) for the entropy evolutions shown in Figure 1 and Figure 2, where *δS*^(*c*)^ is the standard deviation of *S*^(*c*)^, for *t* > 100. Linear regression fit with slope −1.03 ± 0.09 (R^2^ = 0.99).

In Figure 3 we plot ln*ΔS*^(*c*)^ versus ln(*c*) for the entropy evolutions shown in Figure 1 and Figure 2. A linear regression fits the data with a straight line of slope −2.07 ± 0.09 (R^2^ = 0.99). In Figure 4 we further plot ln*δS*^(*c*)^ versus ln(*c*), where *δS*^(*c*)^ is the standard deviation or noise range of *S*^(*c*)^, after a state of virtual equilibrium, $S_{equil}^{\left(c\right)}$, has been reached, which occurs for *t* > 100 in this particular example. A linear regression fits the data in Figure 4 with a straight line of slope −1.03 ± 0.09 (R^2^ = 0.99). The two plots in Figure 3 and Figure 4 thus confirm the general predictions of power laws theoretically derived in Equations (8) and (10), predicting exponents of −2 and −1, respectively, for two-dimensional systems (*d* = 2). In Figure 3 and Figure 4 we further show the c*ell size limit* that corresponds to a single cell comprising the entire system (*c* = 100 in this example). In that case, the entropy is a constant; hence, Equations (8) and (10) are no longer applicable.

**Figure 5:**
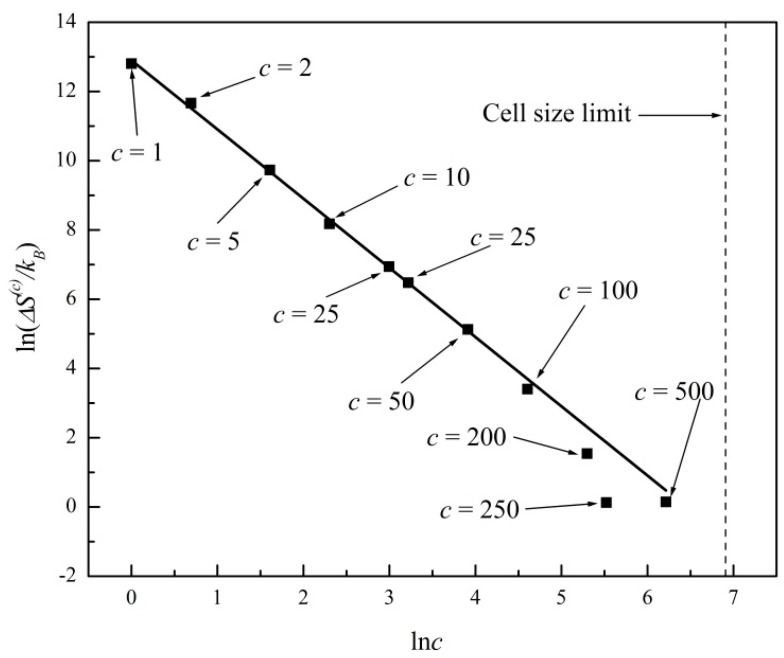
Plot of ln*ΔS*^(*c*)^ versus ln(*c*) for a 1000 × 1000 site lattice gas system. Entropy gaps for *c* > 50 are not included in the linear regression fit, yielding a slope of −1.99 ± 0.03 (R^2^ = 1.00).

**Figure 6:**
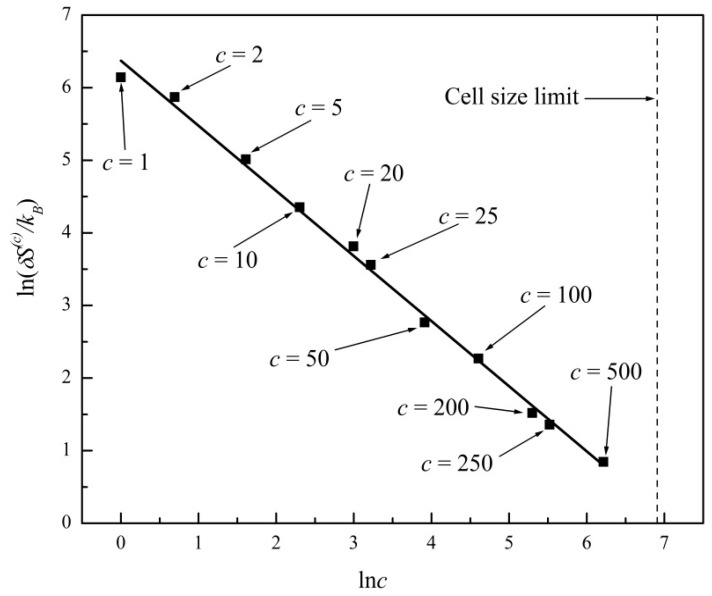
Plot of ln*δS*^(*c*)^ versus ln(*c*) for a 1000 × 1000 site lattice gas system. Linear regression fit with slope −0.90 ± 0.02 (R^2^ = 1.00), including all *c* values up to *c* = 500.

We observed virtually the same behavior for ln*ΔS*^(*c*)^ and *δS*^(*c*)^ for lattices of many different sizes. For example, in Figure 5 and Figure 6 we report results for a 1000 × 1000 site lattice. Again, initially we place one particle on each site, with the 10^6^ initial velocity directions randomly selected. As was already evident in Figure 1 and Figure 2, as *c* increases, the size of the fluctuations decreases and the corresponding maxima, $S_{max}^{\left(c\right)}$, asymptotically approaches an upper bound. In particular, for the 1000 × 1000 site lattice system, the entropy gap becomes quite small only for *c* > 50. In fact, for *c* > 50, the entropy fluctuates in a rather discretized manner, visiting a relatively small subset of values, all of them lying within a narrow range. (See the curve corresponding to *c* = 50 in Figure 2: the tendency of the system toward lower entropies is not apparent there. In fact, the probability of the system revisiting states with $S^{\left(c\right)}=S_{max}^{\left(c\right)}$ is far from negligible in this case.) Thus, it is not surprising that in Figure 5, for *c* > 100, the trend predicted by Equation (8) is not as closely reproduced as it is when *c* is smaller. For that reason, in Figure 5, we did not include entropy gaps for *c* > 50 in the linear fitting that yields the −1.99 ± 0.03 slope of the straight line (R^2^ = 1.00). In Figure 6 we plot the corresponding noise ranges, for which a linear fitting produces a slope of −0.90 ± 0.02 (R^2^ = 1.00), this time including all *c* values up to *c* = 500. From these and many more observations, we have concluded that for wide ranges of coarse graining, the power laws expressed by Equations (8) and (10) describe quite well the essence of coarse-grained entropy gaps and fluctuations when the system has achieved a state of virtual equilibrium.

## 4. Conclusions

We have demonstrated that a finely coarse-grained entropy of a system in a state of effective equilibrium fluctuates over time within a relatively narrow range below the theoretically possible, but practically unattainable, maximum entropy. This fact does not contradict general statements made to the effect that entropy is expected to maintain its maximum value in equilibrium and only quite exceptionally, if ever practically, may it fluctuate substantially below that maximum: see, for instance, Figure 1 on page 31 of Reference [13]. Such statements are generally correct, as long as one recalls that they refer exclusively (i) to the macroscopic entropy in the thermodynamic limit and (ii) to time scales of the order of an immeasurably long Poincaré recurrence time. A maximum entropy formalism may even be elevated to the status of a logical principle [14,15,16]. Our coarse graining approach is complementary, referring to a much more refined and microscopic view of entropy. In fact, that view is consistent with a general perspective on entropy as dependent not just on microscopic states, but also on reference classes corresponding to the relative ability of each observer to identify microscopic states for each system [17]. The relevance of coarse graining in studies of entropy is ever increasing, corresponding to greater and greater possibilities of probing systems at finer and finer microscopic scales, due to major advances in nanotechnology [10,11,12,18]. Power laws regulating coarse graining of entropy, such as those that we have demonstrated in this paper, are expected to more deeply and generally illuminate such phenomena.
